# Analysis of the efficacy and related factors of ventriculoperitoneal shunt for AIDS with cryptococcal meningitis

**DOI:** 10.3389/fsurg.2022.942506

**Published:** 2022-08-26

**Authors:** Zhaohui Chai, Yikai Shou, Rajneesh Mungur, Jiangbiao Gong, Peidong Zheng, Jiesheng Zheng

**Affiliations:** ^1^ Department of Neurosurgery, Hangzhou TCM Hospital Affiliated to Zhejiang Chinese Medical University, Hangzhou, China; ^2^ School of Basic Medical Sciences, Hangzhou Normal University, Hangzhou, China; ^3^ Department of Neurosurgery, The First Affiliated Hospital, College of Medicine, Zhejiang University, Hangzhou, China

**Keywords:** cryptococcal meningitis, CM, increased intracranial pressure, ICP, VPS, AIDS

## Abstract

**Background:**

*Cryptococcus neoformans* is an opportunistic pathogen, which is more common in patients with AIDS. Increased intracranial pressure (ICP) is an important complication of cryptococcal meningitis (CM) and affects the therapeutic effect of CM.

**Objective:**

To evaluate the effect and treatment for the management of ventriculoperitoneal shunt (VPS) in the treatment of AIDS complicated with CM and to analyze the factors associated with VPS and the indices affecting the outcome of CM patients.

**Methods:**

A retrospective case study was conducted on patients with CM treated in the First Affiliated Hospital of Zhejiang University School of Medicine from 2011 to 2019. The Chi-square test was used for categorical variables and the Student’s *t*-test was used for continuous variables. Multivariable analysis of baseline factors related to VPS placement was performed with stepwise logistic regression analysis, factors associated with the outcome of these patients were studied by Cox regression analysis, and Kaplan–Meier survival curves were constructed to assess the outcome of patients.

**Results:**

There were 96 patients with AIDS complicated with CM. VPS had a great effect on the patients, especially those with ICP > 350 mmH_2_O. The outcome, including the mortality rate and modified Rankin scale (MRS) score of these patients, significantly improved after the placement of VPS. The karnofsky performance status (KPS) scores of patients whose ICP > 350 mmH_2_O improved from 39.3 ± 21.3 at baseline to 88.7 ± 26.9 at 3 months after VPS, better than those without VPS. Multivariable analysis showed that visual impairment (OR, 0.026; 95% CI, 0.001, 0.567; *P* = 0.021) and ICP > 350 mmH_2_O (OR, 0.026; 95% CI, 0.002, 0.293; *P* = 0.003) were related elements with the placement of shunt, and KPS score (HR, 0.968; 95% CI, 0.943, 0.993; *P* = 0.013) and ICP > 350 mmH_2_O (HR, 2.801; 95% CI, 1.035, 7.580; *P* = 0.043) were indices of the outcome of AIDS patients with CM. For patients with ICP > 350 mmHg, Kaplan–Meier analysis showed that the 3-year outcome of patients with VPS was better than that of patients without VPS (*P* = 0.0067).

**Conclusion:**

VPS was associated with better 3-year survival rates, and postshunt placement complications like infections were rare. The identification of factors related to VPS in the initial diagnosis of CM can contribute to more active management and improve the outcome.

## Introduction

*Cryptococcus neoformans* is a common conditional pathogen and a common cause of fungal meningitis. The infection rates of *C. neoformans* in AIDS patients are 23% and 48% (1). In many cases, the outcome of this opportunistic infection is not satisfactory. Even if some patients take antifungal therapies, the mortality rate is still high, ranging from 30% to 50% (2). Even with effective antiretroviral therapy (HAART), cryptococcal meningitis (CM) kills 15% of patients every year (3). Therefore, timely and effective treatment should be given to patients with CM in order to improve the outcome of patients with AIDS.

The main symptoms of CM are headache, fever, nausea, vomiting, visual and hearing impairment, and changes in mental state (3). In addition, increased intracranial pressure (ICP) is an important complication, which may lead to disability or even death due to cerebral hernia if not treated in time (2). In patients with ICP, frequent lumbar puncture is required to reduce the pressure and release symptoms. Dehydration drugs such as mannitol and acetazolamide have no obvious effect on reducing intracranial pressure, and the adverse effect of the disturbance of electrolytes limits the application, especially for acetazolamide (4). Uncontrolled increased ICP is defined as an extremely high opening intracranial pressure (>350 mmH_2_O) and failure to control increased ICP symptoms through frequent lumbar punctures and other medical procedures such as glucocorticoids, mannitol, or acetazolamide (5). For a small number of patients, lumbar drainage (6), ventricular drainage (7), Ommaya tube implantation (8), and ventriculoperitoneal shunt (VPS) (9–12) are required to reduce intracranial pressure. But in resource-limiting areas, where most AIDS-related CM cases are reported, the availability of these procedures is lacking. It is difficult to identify patients who require invasive procedures. Studies have shown that VPS can significantly improve the outcome of such patients (13). Therefore, we studied the factors related to VPS in AIDS patients complicated with CM, the effect of VPS on the outcome of patients with AIDS complicated with CM, and the factors related to the outcome, in order to have a more comprehensive understanding of patients who are suitable for VPS.

## Materials and methods

### Subjects

We conducted a retrospective observational cohort study of patients with AIDS complicated with CM. The included cases were diagnosed in the First Affiliated Hospital of Zhejiang University School of Medicine from January 2011 to September 2019. CM is defined as *C. neoformans* discovered in at least one cerebrospinal fluid (CSF) culture, *C. neoformans* capsule antigen positive, cryptococcal ink negative staining positive, and typical CSF characteristics and clinical manifestations. The manifestation of the CSF in patients with CM is consistent with that in patients with lymphocytic meningitis. Generally, the number of white blood cells is less than 150 × 10^6^/L; the protein levels increase and those of glucose decrease. The protocol was approved by the Ethics Committee of the First Affiliated Hospital of Zhejiang University School of Medicine. In order to avoid interfering with the statistical results, the exclusion criteria were as follows: (1) Accompanied by other central nervous system infections; (2) Recurrent cryptococcal meningitis; (3) Sequelae of nervous system injury caused by previous head injury, cerebral infarction, or intracranial hemorrhage; (4) VPS was performed in other hospitals; (5) Implanted Ommaya tube or performed ventricular drainage after diagnosis in our hospital.

### Methods

According to the above criteria, 96 cases were included, which were divided into VPS group and non-VPS group according to the performance or non-performance of the shunt procedure. A total of 30 patients (31.3%) underwent VPS after diagnosis, of which 27 were males and 3 were females, with an average age of 34.7 ± 9.5 years. A total of 66 patients (68.7%) did not undergo VPS, of which 65 were males and 1 was a female, with an average age of 38.8 ± 11.8 years. VPS was undertaken in all patients using a Medtronic® system. We analyzed the data of patients with CM at the time of initial diagnosis in order to reduce the bias caused by physicians’ different preferences for lumbar puncture and shunt. The clinical features of the two groups and the characteristics of patients with ICP > 350 mmH_2_O were compared. The factors associated with VPS, the difference of outcome between the two groups, and the indices related to the outcome were analyzed. Improvement is defined as a reduction of clinical symptoms and signs, and a good outcome is defined as a patient who is still alive, with no symptoms, or with only a few symptoms, and is able to take care of himself/herself. This corresponds to a patient with a modified Rankin scale (MRS) score <2. A poor outcome is defined as the death of a patient or the retention of severe symptoms and thus unable to take care of himself/herself, and this corresponds to a patient with a MRS score ≥2. Hydrocephalus was diagnosed as a dilation of the temporal horn of the lateral ventricle, without significant brain atrophy, and/or an Evan's ratio of >0.30 on the initial and/or subsequent CT or MRI. Evan's ratio is the ratio of the width of the bilateral frontal horn of the ventricle to the maximum biparietal diameter.

### Statistical analysis

The clinical results and biochemical indices were expressed as mean ± standard deviation. The continuous variables such as age, days of diagnosis of AIDS on admission, time from onset to operation (days), karnofsky performance status (KPS) score, CSF nucleated cells, CSF glucose, CSF chloride, CSF protein, and CD4^+^T-cell count were tested by using independent samples. Pearson variance analysis was used to analyze the classified variables such as sex, clinical manifestation, and outcome including the mortality rate and MRS score. Logistic regression analysis was used to analyze the factors related to VPS at the initial diagnosis of CM, and Cox regression analysis was used to analyze the indices related to outcome. A value of *P* < 0.05 was considered to have significant difference. The survival rate was evaluated by Kaplan–Meier analysis. All statistical analyses were conducted using SPSS software, version 23.0.

## Results

### Baseline data of patients

The clinical manifestations of the patients mainly include headache, fever, cranial nerve paralysis, and mental state changes. There was no significant difference in these aspects between the two groups (*P* > 0.05). The KPS score of the VPS group was lower than that of the non-VPS group. The ICP of patients in the VPS group was 404.2 ± 39.8, while the ICP of patients in the non-VPS group was 301.7 ± 105.0, and there was a significant difference between the two groups (*P *< 0.001). The *C. neoformans* count in patients with VPS was higher than that in patients without VPS. The incidence of neck stiffness in the VPS group (70.0%) was higher than that in the non-VPS group (47.0%) (*P* < 0.05). The incidence of convulsions in the VPS group (20.0%) was higher than that in the non-VPS group (1.5%) (*P* < 0.05). There were 11 cases of visual impairment and 2 cases of hearing impairment, and there was significant difference between the two groups (*P* < 0.05). The average length of stay in the non-VP shunt group was shorter than that in the VP shunt group, but there was no significant difference between the two groups (Table 1). The ICP significantly decreased after VPS (**Supplementary Figure S1**).

**Table 1:** Comparison of baseline data between the VPS group and the non-VPS group.

Characteristics	Non-VPS group (*n* = 66)	VPS group (*n* = 30)	*P*-value
Gender (M/F)	65/1	27/3	0.054
Age, mean (SD)	38.8(11.8)	34.7(9.5)	0.100
Days diagnosed AIDS before admission, mean (SD)	284.1(664.8)	96.8(392.3)	0.159
Headache, *n* (%)	53(80.3)	26(86.7)	0.393
Fever, *n* (%)	41(62.1)	18(60.0)	0.853
Nausea, *n* (%)	27(40.9)	23(76.7)	0.001
Emesis, *n* (%)	21(31.8)	21(70.0)	<0.001
Visual symptoms, *n* (%)	2(3.0)	9(30.0)	<0.001
Hearing symptoms, *n* (%)	0(0.0)	2(6.7)	0.028
Cranial nerve palsy, *n* (%)	2(3.0)	1(3.3)	0.891
Mental status change, *n* (%)	9(13.6)	4(13.3)	0.968
Neck stiffness, *n* (%)	31(47.0)	21(70.0)	0.036
Convulsion, *n* (%)	1(1.5)	6(20.0)	0.001
KPS, mean (SD)	49.2(22.2)	38.7(21.2)	0.033
Evans’ ratio	0.25(0.03)	0.26(0.03)	0.211
ICP, (mmH_2_O), mean (SD)	301.7(105.0)	404.2(39.8)	<0.001
Ink stain, *n* (%)	55(83.3)	29(96.7)	0.088
CSF culture, *n* (%)	46(69.7)	26(86.7)	0.174
Cryptococcus counts, (10^6^/L), mean (SD)	2.6(1.4)	3.5(1.8)	0.023
CSF WBC, (10^6^/L), mean (SD)	34.4(95.4)	12.0(32.4)	0.217
Glucose, mmol/l, mean (SD)	2.6(0.9)	3.1(1.1)	0.019
Protein, g/l, mean (SD)	0.7(0.6)	0.6(0.4)	0.160
Chloride, mmol/l (SD)	117.0(6.3)	118.0(5.1)	0.458
CD4, (10^6^/L) (SD)	20.9(37.0)	15.5(19.5)	0.460
CD4/CD8, mean (SD)	0.10(0.09)	0.05(0.05)	0.007
Hospital stay, mean (SD)	38.9(37.8)	60.1(67.4)	0.055

SD, standard deviation; CSF WBC, cerebrospinal fluid white blood cell; VPS, ventriculoperitoneal shunt.

### Comparison of patients with ICP > 350 mmH_2_O

**Figure 1:**
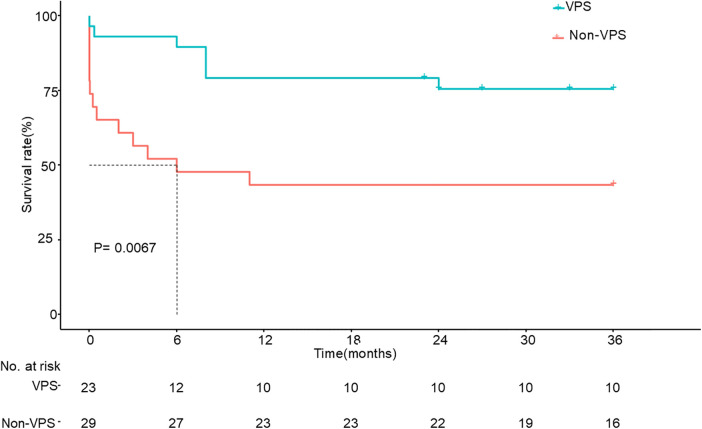
Kaplan–Meier survival analysis between the two groups whose ICP > 350 mmH_2_O. ICP, increased intracranial pressure; VPS, ventriculoperitoneal shunt.

Nausea, vomiting, and visual impairment in patients with VPS were more obvious than those without VPS. Among the patients who underwent VPS, 5 (17.2%) had convulsions, while those without VPS had no convulsions, and there was a significant difference between the two groups (*P* < 0.05). At the same time, for patients with ICP > 350 mmHg, the short-term and long-term outcomes (mortality rates, MRS scores) of patients with VPS were better than those of patients without VPS. The KPS score at baseline of patients with VPS was 39.3 ± 21.3, while it was 39.6 ± 18.5 for patients without VPS (*P* = 0.965), and there were no distinct differences between the two groups. The KPS score at 3 months of patients with VPS was 88.67 ± 26.92, while it was 58.26 ± 46.96 for patients without VPS (*P* = 0.012). Table 2 shows a comparison between the two groups. Figure 1 shows the Kaplan–Meier analysis of the 3-year outcome of the two groups.

**Table 2:** Comparison of basic data between the VPS group and the non-VPS group whose ICP > 350 mmH_2_O.

Characteristics	Non-VPS group (*n* = 23)	VPS group (*n* = 29)	*P*-value
Gender (M/F)	23/0	26/3	0.112
Age, mean (SD)	38.9(12.5)	34.6(9.7)	0.178
Days diagnosed AIDS before admission, mean (SD)	283.6(729.8)	98.0(399.0)	0.258
Headache, *n* (%)	20(87.0)	25(86.2)	0.849
Fever, *n* (%)	13(56.5)	18(62.1)	0.707
Nausea, *n* (%)	10(43.5)	22(75.9)	0.015
Emesis, *n* (%)	8(34.8)	20(69.0)	0.013
Visual symptoms, *n* (%)	0(0.0)	9(31.0)	0.002
Hearing symptoms, *n* (%)	0(0.0)	2(6.9)	0.183
Cranial nerve palsy, *n* (%)	0(0.0)	1(3.4)	0.351
Mental status change, *n* (%)	4(17.4)	3(10.3)	0.460
Neck stiffness, *n* (%)	18(78.3)	20(69.0)	0.453
Convulsion, *n* (%)	0(0.0)	5(17.2)	0.036
KPS at baseline, mean (SD)	39.6(18.5)	39.3(21.3)	0.965
KPS at 3 months, mean (SD)	58.3(47.0)	88.7(26.9)	0.012
Ink stain, *n* (%)	22(95.7)	28(96.6)	0.867
CSF culture, *n* (%)	16(69.6)	26(89.7)	0.116
Cryptococcus counts, (10^6^/L), mean (SD)	2.9(1.5)	3.5(1.8)	0.272
CSF WBC, 10^6^/L (SD)	15.7(31.5)	12.4(32.9)	0.716
Glucose, mmol/l (SD)	2.7(0.7)	3.1(1.1)	0.110
Protein, g/l, mean (SD)	0.5(0.2)	0.5(0.4)	0.457
Chloride, mmol/l (SD)	115.7(5.6)	118.1(5.2)	0.122
CD4, (10^6^/L) (SD)	10.1(14.8)	15.6(19.9)	0.309
CD4/CD8, mean (SD)	0.08(0.06)	0.05(0.05)	0.124
Hospital stay, mean (SD)	54.3(57.0)	61.6(68.0)	0.690
Outcome at 1 month (Mortality rate), *n* (%)	8(34.8)	1(3.4)	0.003
Outcome at 3 months (Mortality rate), *n* (%)	9(39.1)	2(6.9)	0.004
Outcome at 6 months (Mortality rate), *n* (%)	11(47.8)	2(6.9)	0.005
Good MRS score at 3 months, *n* (%)	14(60.9)	26(89.7)	0.014
Good long-term MRS score, *n* (%)	10(43.5)	22(75.9)	0.017

MRS, modified Rankin scale; SD, standard deviation; CSF WBC, cerebrospinal fluid white blood cell; VPS, ventriculoperitoneal shunt.

### Postoperative complications

There were two cases of shunt infection, no ventriculitis, two cases of proximal catheter obstruction or displacement, no tube rupture, seven cases of excessive drainage, one case of too little drainage, no distal tube obstruction, no peritonitis, and two cases of abdominal shunt displacement. Of the two patients with shunt infection, one patient underwent shunt adjustment operation 2 months after operation, and the other removed the shunt tube approximately 1 year after operation. These two patients had a good outcome. There were two patients with proximal catheter obstruction or displacement and two others with shunt abdominal end displacement, and all of them underwent shunt adjustment. One patient with proximal catheter obstruction had a poor outcome, and the rest had a good outcome. The pressure of the patients with excessive drainage returned to normal after pressure regulation, and the outcome was good. One patient with too little drainage was complicated with proximal catheter obstruction and the outcome was poor.

### Multivariate analysis

In logistic regression analysis, visual impairment (OR, 0.026; 95% CI, 0.001, 0.567; *P* = 0.021) and ICP > 350 mmH_2_O (OR, 0.026; 95% CI, 0.002, 0.293; *P* = 0.003) were factors related to the placement of VPS (Table 3). In Cox regression analysis, KPS score (HR, 0.968; 95% CI, 0.943, 0.993; *P* = 0.013) and ICP > 350 mmH_2_O (HR, 2.801; 95% CI, 1.035, 7.580; *P* = 0.043) were indices related to the outcome of patients with AIDS complicated with CM (Table 4).

**Table 3:** Logistic regression analysis of baseline factors associated with shunting.

Variables	Univariate		Multivariate	
	OR (95% CI)	*P*-value	OR (95% CI)	*P*-value
Days diagnosed AIDS before admission	0.999(0.998,1.000)	0.196	0.999(0.997,1.001)	0.289
Visual symptoms	0.066(0.013,0.332)	0.001	0.026(0.001,0.576)	0.021
KPS	0.977(0.955,0.999)	0.038	0.993(0.962,1.025)	0.654
Cryptococcus counts	1.409(1.034,1.919)	0.030	1.322(0.859,2.036)	0.204
ICP > 350 mmH_2_O	0.018(0.002,0.144)	<0.001	0.026(0.002,0.293)	0.003
Neck stiffness	0.380(0.152,0.951)	0.039	0.993(0.212,4.653)	0.993

OR, odds ratio; CI, confidence interval; ICP, increased intracranial pressure.

**Table 4:** Cox analysis of baseline factors associated with prognosis.

Variables	Univariate		Multivariate	
	HR (95% CI)	*P*-value	HR (95% CI)	*P*-value
Headache	0.975(0.335,2.836)	0.963	0.727(0.239,2.213)	0.575
Visual symptoms	1.074(0.279,4.134)	0.917	1.122(0.252,5.000)	0.880
Neck stiffness	4.312(1.725,10.778)	0.002	2.096(0.771,5.695)	0.147
KPS	0.956(0.932,0.981)	0.001	0.968(0.943,0.993)	0.013
ICP>350 mmH_2_O	4.432(1.836,10.699)	0.001	2.801(1.035,7.580)	0.043

HR, hazard ratio; CI, confidence interval; ICP, increased intracranial pressure.

## Discussion

Even with the widespread use of highly active antiretroviral therapy (HAART), CM can kill approximately 15% of AIDS patients every year (14, 15). For AIDS patients with acute CM, it is important to realize that the deterioration of clinical conditions is caused by an increase of ICP. Graybill et al. (16) and Pappas et al. (17) indicate that the initial ICP of 50% AIDS patients complicated with CM is >250 mmH_2_O. Dehydration drugs such as mannitol and acetazolamide have the effect of reducing intracranial pressure, but the effect is not significant, especially for acetazolamide, which can lead to a decrease of blood bicarbonate and an increase of blood chlorine, as well as some other adverse events. Many guidelines recommend continuous lumbar puncture for treatment, but it is difficult to quickly bring down ICP to normal level (18). VPS is a good method to control ICP in patients with CM. However, the indication of VPS in AIDS patients complicated with CM and the indices associated with the outcome of VPS are unclear. It is generally believed that VPS should be performed in patients with hydrocephalus, but most patients are often not associated with an enlarged ventricular system (19, 20). A total of 96 patients in our study were not associated with ventricular enlargement, and while some patients underwent VPS, the outcome significantly improved. Therefore, we should seek the help of a neurosurgeon even without ventriculomegaly.

Identifying patients when they have AIDS complicated with CM diagnosis and who may require VPS may aid in improving the outcome. Baddley et al. (11) showed that ICP > 300 mmH_2_O and hydrocephalus were associated with VPS in CM patients. Our multivariable analysis showed that visual impairment and ICP > 350 mmH_2_O were associated with VPS in AIDS patients with CM.

Liang Wu et al. (13) analyzed 128 patients with AIDS complicated with CM, among which 24 underwent shunt surgery (5 with VPS and 19 with lumboperitoneal shunt). The author concluded that the outcome of patients undergoing shunt surgery was better than that of patients without shunt surgery.

Chih-Wei Hung et al. (5) analyzed 180 HIV-negative patients with CM and concluded that convulsions were associated with poor outcome and prolonged hospital stay. Anekthananon et al. (21) analyzed 140 cases of HIV-infected patients complicated with CM and concluded that factors associated with poor outcome at 14 days included high cryptococcal antigen titer (≥1:1024), low body weight, and low CSF white blood cell and factors associated with poor 70-day outcome included high cryptococcal antigen titer (≥1:1024) and low KPS score. However, prognostic indicators of VPS in these patients are lacking. Our study found that a low KPS score and ICP > 350 mmH_2_O were associated with poor outcome in patients with AIDS complicated with CM. Patients with low KPS scores may have progressive AIDS, which may lead to worse clinical outcomes. Uncontrollable increased ICP is defined as an extremely high pressure (>350 mmH_2_O) and failure to control symptoms with frequent lumbar punctures and the use of other medication such as mannitol, acetazolamide, or corticosteroids (5). In our study, the presence of ICP > 350 mmH_2_O indicates a poor outcome.

Although some patients with ICP > 350 mmH_2_O had a good outcome without VPS, in most patients with ICP > 350 mmH_2_O, VPS could significantly improve the outcome. Kaplan–Meier analysis showed that the 3-year survival rate of patients with VPS was significantly better than that of patients without VPS.

Common complications of VPS include obstruction, catheter rupture, dysfunction, infection (fungal infection or secondary infection), and excessive drainage. Other rare complications include subdural and intracranial hematoma, intestinal perforation, abdominal pseudocyst, hernia, and epilepsy (22). Subdural and intracerebral hematomas often occur in the cerebral ventricle, and atrophy of the brain and decompression of the ventricular system occur too quickly (23). If there is a high level of protein in the CSF, the risk of obstruction increases (24). A reasonable placement of the shunt tube can reduce the possibility of shunt blockage. If obstruction occurs, the shunt requires to be adjusted or even replaced. The concept of asepsis should be strictly borne in mind by the surgeon during the whole operation, and prophylactic use of antibiotics after shunt placement can reduce secondary infection. If infection occurs, it is necessary to remove the shunt tube and carry out anti-infective treatment. After the condition becomes stable, the tube should be placed again.

This study has several limitations. Although this is a large series of AIDS patients with CM managed with VPS, we were limited in our multivariable analyses due to only 30 shunt events. It is likely that we were unable to include some potentially important variables into the final multivariable model. This is a retrospective study, and therefore, it is subject to bias of some unmeasured factors. Also, during this study, the indication or frequency of lumbar punctures was not standardized, and thus we could not get more details about the alteration of ICP and the constituents of the CSF. Some scores, such as the glasgow coma scale score, were not evaluated in each patient, so this part of the data is missing. Our data may not be applicable in resource-limited settings where shunting is not available.

## Conclusion

In summary, VPS was associated with better 3-year survival rates and good outcome, and postshunt placement complications like infections were rare as well. An identification of prognostic factors in the diagnosis of AIDS with CM and factors associated with VPS can improve the outcome of such patients. We opine that an analysis of the initial clinical data at the time of diagnosis of CM is very appropriate because it can reduce the impact caused by indications. For example, the timing and choice of shunt are related to the preferences of neurosurgeons and infectious disease surgeons. Given the small sample size of our study, more studies on VPS in patients with AIDS and CM are needed.

## Data Availability

The raw data supporting the conclusions of this article will be made available by the authors, without undue reservation.
