# Supplementary figures and images for: Analysis of the efficacy and related factors of ventriculoperitoneal shunt for AIDS with cryptococcal meningitis
Source: Front Surg. 2022 Aug 26;9:942506. doi: 10.3389/fsurg.2022.942506 (PMC9479539; doi:10.3389/fsurg.2022.942506)

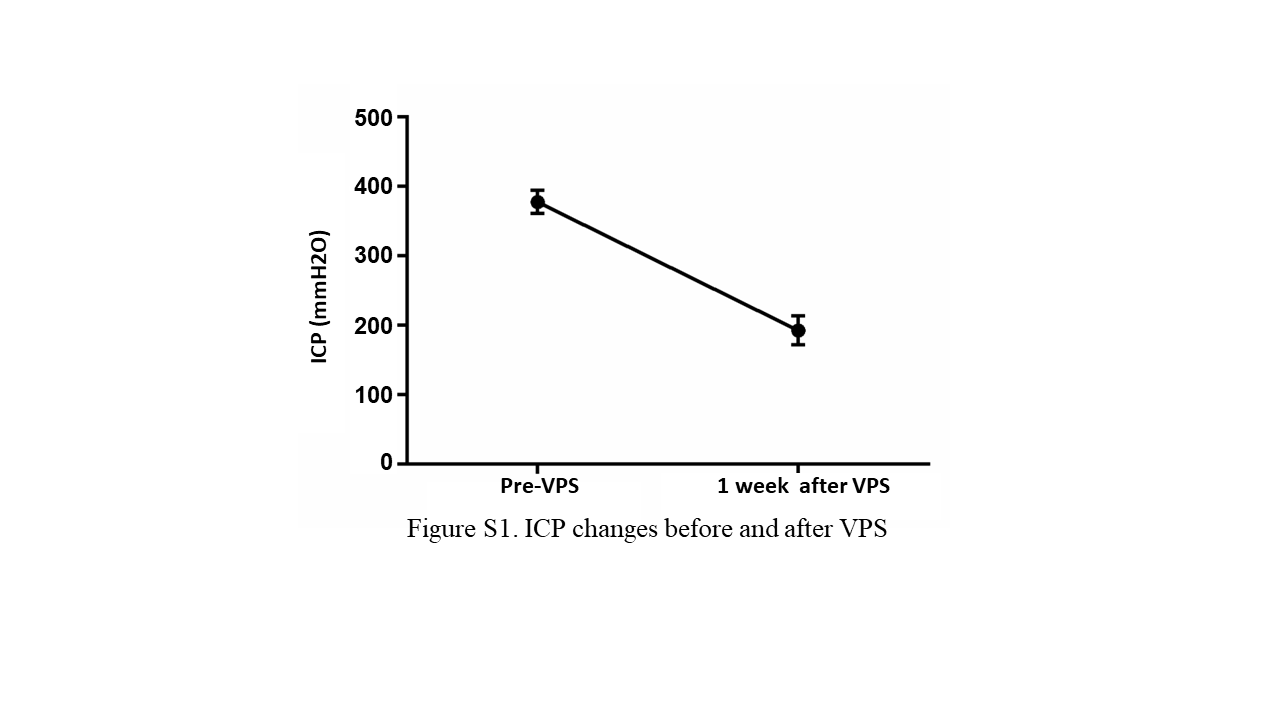

Supplement: Supplementary file 1 [file Image_1_v1.tif]
